# Incidence and predictors of AF recurrence during long-term follow-up of patients after PF ablation for atrial fibrillation

**DOI:** 10.1093/europace/euag057

**Published:** 2026-03-23

**Authors:** Anna-Sophie Eberl, Martin Manninger, Ursula Rohrer, Laura Stix, Stefan Kurath-Koller, Katharina Gölly, Martin Benedikt, Egbert Bisping, Peter Lercher, Andreas Zirlik, Daniel Scherr

**Affiliations:** Division of Cardiology, Department of Medicine, Medical University of Graz, Austria; Division of Cardiology, Department of Medicine, Medical University of Graz, Austria; Division of Cardiology, Department of Medicine, Medical University of Graz, Austria; Division of Cardiology, Department of Medicine, Medical University of Graz, Austria; Division of Paediatric Cardiology, Department of Paediatrics & Adolescence Medicine, Medical University of Graz, Austria; Division of Cardiology, Department of Medicine, Medical University of Graz, Austria; Division of Cardiology, Department of Medicine, Medical University of Graz, Austria; Division of Cardiology, Department of Medicine, Medical University of Graz, Austria; Division of Cardiology, Department of Medicine, Medical University of Graz, Austria; Division of Cardiology, Department of Medicine, Medical University of Graz, Austria; Division of Cardiology, Department of Medicine, Medical University of Graz, Austria

**Keywords:** Ablation, Atrial fibrillation, Electroporation, Pulmonary vein isolation, Pulsed field ablation

## Abstract

**Aims:**

Pulsed field ablation (PFA) is an established technology for pulmonary vein isolation (PVI) in patients with atrial fibrillation (AF). However, data on long-term outcome remain scarce. With this work, we aim to contribute valuable long-term data on catheter ablation with PFA.

**Methods and results:**

We conducted a retrospective analysis of our single-centre data of 339 patients (63% paroxysmal AF, 37% persistent AF), as well as of 55 redo procedures in patients, who underwent first PVI with a pentaspline PFA catheter. During the median follow-up (FUP) of 752 (391–1486) days, 34% patients (*n* = 116) experienced arrhythmia recurrence after a blanking period of 90 days, with a median time to recurrence of 218 (90–1161) days. Multivariate analysis showed electrical cardioversion at the end [HR 1.97 (95% CI 1.17–3.33), *P* = 0.011] and AF at the beginning of the procedure [HR 1.73 (95% CI 1.04–2.88), *P* = 0.034] being independently associated with a higher risk of arrhythmia recurrence. Additional anterior flower applications were protective in the univariate (*P* = 0.025) analysis. Atrial tachycardia (AT) was present in 16, 37, 0, and 0% after the first, second, third, and fourth procedure, respectively. In 55 analysed redo procedures, 104/221 veins (47%) were reconnected (0/1/2/3/4 reconnected veins: 9%/31%/27.3%/27.3%/5.4%). Analysis of multiple procedure outcome estimates improved long-term arrhythmia-free survival, with an overall success rate of 86% after ≥2 procedures.

**Conclusion:**

PV reconnections are frequent in patients presenting for repeat ablations, especially at the anterior PV aspect. A multiple procedure approach estimates arrhythmia-free survival in 86% of patients. Procedures with additional anterior lesions at the right pulmonary veins (RPVs) could be protective for recurrences.

## Graphical Abstract

**Graphical Abstract:**
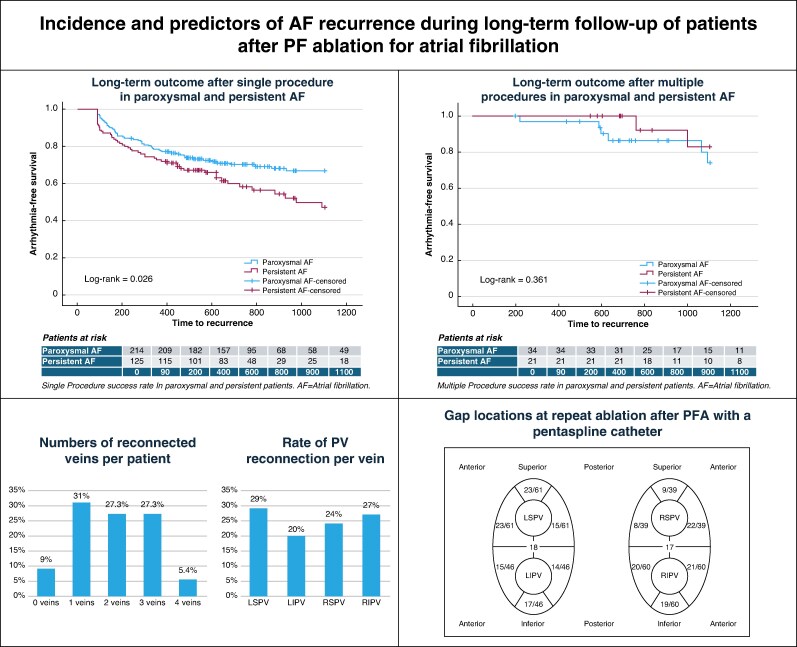

What’s new?

- Single-procedure success rates after pulmonary vein isolation (PVI) with PFA remain comparable with RF procedures even after a median follow-up of more than two years (PAROX = 71%; PERS = 58%). Multiple procedure success rates are even higher (PAROX = 82%; PERS = 90%).
- Only a few atrial tachycardias occur as recurrent arrhythmias after the first PVI (16%), whereas this becomes more common after the second procedure (37%).
- PVI durability remains an issue with high reconnection rates during redo procedures. Two additional anterior flower applications at the RPVs seem to prevent PV reconnections.

## Introduction

Atrial fibrillation (AF) is the most common sustained arrhythmia, resulting in significant costs for the health care system.^1^ As catheter ablation (CA) is a cornerstone of rhythm control management, various methods have been developed during the past years.^2^ Pulsed field ablation (PFA) is an established quick and safe method with similar success rates to thermal ablation strategies.^3–5^ This method locally disturbs the electric field by applying short electrical pulses with high energy, creating irreversible micropores in the cell membrane of cardiomyocytes, when reaching a certain threshold. This leads to leakage, unregulated ion inflow and outflow, and ultimately cell death.^6,7^ As cardiac cells appear to have the lowest threshold, they show a relative sensitivity to the pulsed electric field (PEF), while adjacent tissue is less affected, contributing to its promising safety profile.^8,9^

As the pentaspline catheter was the first widely used single-shot PFA device, it has already been evaluated in large registries^4,5,10^ and two randomized controlled trials.^3,11^ However, there is a lack of long-term data on outcome, recurrent arrhythmias, and electrophysiological findings during redo procedures.

## Methods

This study reports on our single-centre experience with PFA for first-do pulmonary vein isolation (PVI) using the Farapulse® system (Boston Scientific). All patient data were obtained from the local registry for patients undergoing CA (ethical approval number: 31-037ex18/19).

### Ablation protocol during PFA procedure

Patients were either on uninterrupted oral anticoagulation for at least 4 weeks or left atrial appendage thrombi were ruled out before the procedure. Preprocedural computed tomography scan to evaluate pulmonary vein (PV) anatomy was performed routinely. Procedures were performed under deep sedation with propofol and fentanyl. We aimed for an activated clotting time (ACT) level above 350 s throughout the procedure. Following ultrasound-guided groin puncture, a quadripolar catheter was placed in the right ventricle (RV) and a decapolar catheter into the coronary sinus (CS). The RV catheter was withdrawn to the right ventricular lumen to avoid previously described perforation,^4,5^ and has been abandoned since early 2025. After TSP, using either a Brockenbrough needle (BRK XS, Saint Jude Medical®), a radiofrequency (RF) needle (NRG Transseptal Needle®, Boston Scientific), or VersaCross® (Boston Scientific), the 13 F steerable Faradrive® sheath and a 31 or 35 mm pentaspline catheter were inserted into the left atrium (LA). A 35 mm catheter was used for PV ostia > 20 mm and/or severe LA dilation.

After administration of 1 mg of atropine, each vein was treated with eight PEF applications per vein (four in basket, four in flower configuration), with five pulses of a pulsed field amplitude of 2.0 kV. Left common ostia (LCO) were treated by cannulating an upper and a lower branch with eight applications each. Additional ablation of a right middle pulmonary vein (RMPV) or a roof vein was performed when not covered during adjacent vein ablation.

Since January 2024 two additional ablations in flower configuration with an anterior twist at both right PVs have been routinely performed (*n* = 84). Posterior wall isolation (PWI) was performed at the operator’s discretion in patients with persistent AF (PersAF), using anchoring lesions at each vein (cannulation of the PV followed by a posterior twist) and overlapping PEF applications across the entire posterior wall, guided by fluoroscopy or 3D electroanatomical mapping (3D-EAM) (EMS; CARTO®, Biosense Webster). This analysis contains no procedure in which Faraview® (Boston Scientific) was used. For additional ablation of the cavotricuspid isthmus (CTI), RF was used. Left atrial mitral isthmus (MI) ablation was performed with either RF or PFA.

In all cases veins were checked for signals and for entrance and exit block to confirm isolation in sinus rhythm (SR) with the catheter placed at the PV ostia. If SR was not restored, electrical cardioversion (ECV) was performed before. If complete isolation was not achieved, additional applications were done accordingly. No oesophageal temperature probe was used throughout all procedures. Since 2024 the groin access for the 13 Fr sheath was closed with the ProStyle® closure system, followed by 4–6 h of bed rest. Antiarrhythmic therapy was routinely continued for 3 months after ablation and was not considered a predictor of recurrence in the analysis. Patients were discharged the same or the following day, if no periprocedural complication was observed.

### Ablation protocol during redo procedure

Patients with symptomatic AT/AF recurrence after a blanking period (BP) of 90 days were offered a redo procedure. Sedation protocol and ACT levels were identical to those in the index procedure. Most redo procedures were performed using RF, with two cases using an alternative PFA system (VARIPULSE®, Biosense Webster) and two cases combining Farapulse® with RF. 3D-EAM was performed in all redo procedures. If voltage mapping showed reconnected veins, the gaps were closed. Afterwards, arrhythmia induction via atrial burst pacing and infusion of isoproterenol was attempted. If additional arrhythmias could be initiated, the operator would perform further ablation at their discretion. The endpoint for any linear lesion was a bidirectional block.

### Data acquisition, FUP, and definition of recurrences

Patients received 24 h Holter ECGs at 3, 6, and 12 months. Additionally, the hospital information system (OpenMEDOCS®, SAP Patient Management) and the cross-state electronic health record (ELGA, ELGA GmbH, Vienna) were scanned for any documented arrhythmia recurrence. Foreign patients were followed up by telephone. Documentation of sustained atrial arrhythmia longer than 30 s was defined as arrhythmia recurrence.

### Statistical analysis

Analysis and comparison were computed in Excel (Microsoft Office 365) and IBM SPSS Statistics for Windows, Version 30. Continuous variables are presented as median and range, while categorical variables are presented as percentages and counts. Event-free survival was estimated using Kaplan–Meier’s method and the log-rank test. For statistical analysis, gaps originating from common ostia [LCO, right common ostium (RCO)] were reassigned to their corresponding anatomical PV location, allowing the analysis to be restricted to the four standard PVs. Predictors of AF recurrence were analysed using a univariate Cox proportional hazard model. Variables with *P* < 0.10 in univariate analysis, as well as those considered clinically relevant, were subsequently included in a multivariable Cox proportional hazards model. A *P*-value of <0.05 was assumed to be significant.

## Results

### Baseline data of the total PFA cohort and long-term FUP

Between June 2021 and June 2024, we performed PVI with PFA in 349 patients at the University Hospital Graz. Ten patients had to be excluded from our analysis, due to loss of follow-up (FUP); thus, 339 patients [63% paroxysmal atrial fibrillation (PAF), 37% PersAF (of which 3.8% long standing persistent; *n* = 13)] were analysed in total (*Table 1*).

**Table 1:** Baseline characteristics before index procedure

	Total cohort (*n* = 339)	Recurrence (*n* = 116)	No recurrence (*n* = 223)	*P*-value (univariate)
**Gender**				*P* = 0.094
Female	31%	38%	27%	
Male	69%	62%	73%	
**Age (year)**				*P* = 0.181
Median (range)	62 (31–85)	63 (32–82)	62 (31–85)	
**LVEF**				
≥50%	91%	91%	91%	*P* = 0.617
49–41%	3%	2.7%	3.5%	*P* = 0.829
40–35%	2.5%	4.5%	1.5%	*P* = 0.288
<35%	3%	1.8%	4%	*P* = 0.455
**LA diameter**				*P* = 0.328
Normal/mild/moderate severe/very severe dilation (≥50 mm)	88.5%	86%	90%	
	11.5%	14%	10%	
**CHADS-VA**				***P*** **=** **0.005**
Median (range)	2 (0–6)	2 (0–6)	1 (0–6)	
**BMI (kg/m^2^)**				*P* = 0.267
Median (range)	27 (15–46)	27 (20–46)	27 (15–46)	
**AF type**				***P*** **=** **0.014**
Paroxysmal	63%	54%	68%	
Persistent	37%	46%	32%	
**Hx of PersAF (months)**				*P* = 0.197
Median (range)	20 (1–240)	24 (1–240)	16.5 (2–156)	
**Anatomical variant**				*P* = 0.485
Normal anatomy	75%	77.6%	74%	
LCO	9%	5%	11%	
RMPV	11%	12%	11%	
LCO + RMPV	2%	1.7%	2.2%	
RCO	0.6%	1.7%	—	
Roof vein	0.3%	—	0.4%	
Others	1.6%	1.7%	1.3%	
**Comorbidities**				
Hypertension	57%	61%	54%	*P* = 0.313
Diabetes	9%	13%	6.3%	***P*** **=** **0.013**
CAD	17%	15.5%	17.5%	*P* = 0.644
Hyperlipidaemia	27%	27.5%	27%	*P* = 0.397
Stroke	7%	8%	6%	*P* = 0.475
Cardiomyopathy	11%	15.5%	9%	***P*** **=** **0.023**
**AAD**	**total: 44%**	**total: 43%**	**total: 45%**	
Class I	17%	16%	18%	—
Class III	27%	27%	27%	—
Class II	Only BB: 44%	Only BB: 45%	Only BB: 43%	—
	73%	75%	71%	
**NT-proBNP (pg/mL)**				*P* = 0.129
Median (range)	222.5 (6–4696)	285 (21–2258)	165 (6–4696)	
**Creatinine (mg/dL)**				*P* = 0.455
Median (range)	1.0 (0.54–2.36)	1.02 (0.54–1.84)	1 (0.62–2.36)	

LVEF, left ventricular ejection fraction; LA diameter, left atrial diameter [measured in parasternal long axis (PLAX) view]; BMI, body mass index; AF, atrial fibrillation; LCO, left atrial common ostium; RMPV, right middle pulmonary vein; RCO, right common ostium; PV, pulmonary vein; CAD, coronary artery disease; AAD, antiarrhythmic drug; —, none.

Values in brackets show range (minimum–maximum)

A total of 84 patients completed the 3-year FUP (median 1239 days). In 98 and 157 patients, 2 years (median 901 days) and at least 1 year (median 526) of FUP are provided, respectively. The median FUP of the full cohort was 752 (391–1486) days.

### Procedural data for index procedure of the total PFA cohort

A 31 mm pentaspline catheter was used in 96% of cases. In 82% of the procedures (*n* = 277), all veins were successfully isolated on the first pass. Acute reconnections requiring further ablation were found in 4.6% (74/1599 veins), while complete PV isolation was achieved in all patients by the end of the procedure. In 25% of patients additional anteriorly located flower applications were given at the right PVs. In addition, CTI ablation was performed in 5.6%, PWI in 2.7%, and MI in 1.2% of patients. The median procedure time was 55 (24–175) minutes, and the median x-ray dose was 12.6 (0.18–110) Gy/cm². Catheter-associated complications occurred in 1.2% (all minor vascular, such as haematoma). Nine out of 339 patients (2.7%) had prolonged atrial asystole longer than 3 s after PFA delivery. More procedure-related data and differences are provided in *Table 2*.

**Table 2:** Procedural data of index procedure

	Total cohort (*n* = 339)	Recurrence (*n* = 116)	No recurrence (*n* = 223)	*P*-value (univariate)
**Deep sedation**	100%	100%	100%	—
**First pass isolation (FPI)**	82% (*n* = 277)	84.5%	80%	*P* = 0.569
**Add. pulses if no FPI**				*P* = 0.953
No. of veins w/o FPI	*n* = 74	*n* = 23	*n* = 51	
LCO	5%	9%	4%	
LSPV	53%	48%	55%	
LIPV	19%	22%	18%	
RSPV	12%	17%	10%	
RIPV	11%	4%	14%	
**PV isolation**	100%	100%	100%	—
**Additional ablation**				
CTI	5.6%	3.4%	6.7%	*P* = 0.168
PWI	2.7%	1.7%	3.1%	*P* = 0.442
Mitral isthmus	1.2%	0.9%	1.3%	*P* = 0.660
Add. Ant. appl. RPVs	25%	12%	31%	***P*** **=** **0.025**
**Pre/post-mapping**	6%	5%	6%	*P* = 0.409
**Use of ICE**	1.8%	2.6%	1.3%	*P* = 0.571
**Catheter size**				*P* = 0.770
31 mm	96%	95%	96.5%	
35 mm	4%	5%	3.5%	
**Catheter-associated complications**				*P* = 0.189
Minor vascular	1.2%	1.7%	0.9%	
Major vascular	—	—	—	
**Vagal responses**	2.7%	2.6%	2.7%	*P* = 0.938
**Procedure duration**				*P* = 0.561
Median (range)	55 (24–175)	60 (27–134)	55 (24–175)	
**X-ray dose**				*P* = 0.418
Median (range)	12.6 (0.18–110)	13.4 (0.18–60)	12.1 (0.81–110)	
**Heart rhythm at the beginning of the proc.**				***P*** **<** **0.001**
SR or pacing	68%	50%	78%	
AF	32%	50%	22%	
**ECV at the end**				***P*** **<** **0.001**
Yes	21.6%	37%	14%	

LCO, left common ostium; LSPV, left superior pulmonary vein; LIPV, left inferior pulmonary vein; RSPV, right superior pulmonary vein; RIPV, right inferior pulmonary vein; RPVs, right pulmonary veins; CTI, cavotricuspid isthmus; PWI, posterior wall isolation; ICE, intracardiac echocardiography; SR, sinus rhythm; AF, atrial fibrillation; ECV, electrical cardioversion; —, none.

Values in brackets show range (minimum–maximum).

### Long-term and multiple procedure outcomes

**Figure 1:**
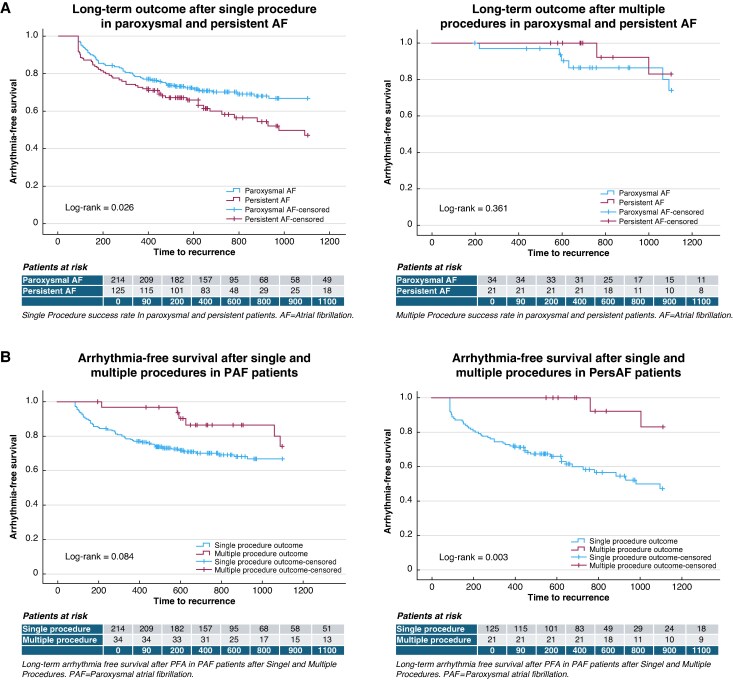
(*A*) Single (left) and multiple (right) procedure outcome in paroxysmal and persistent AF. AF, atrial fibrillation. (*B***)** Outcome in paroxysmal patients according to the single and multiple procedure approach (left). Outcome in persistent patients according to the single and multiple procedure approach (right).

**Figure 2:**
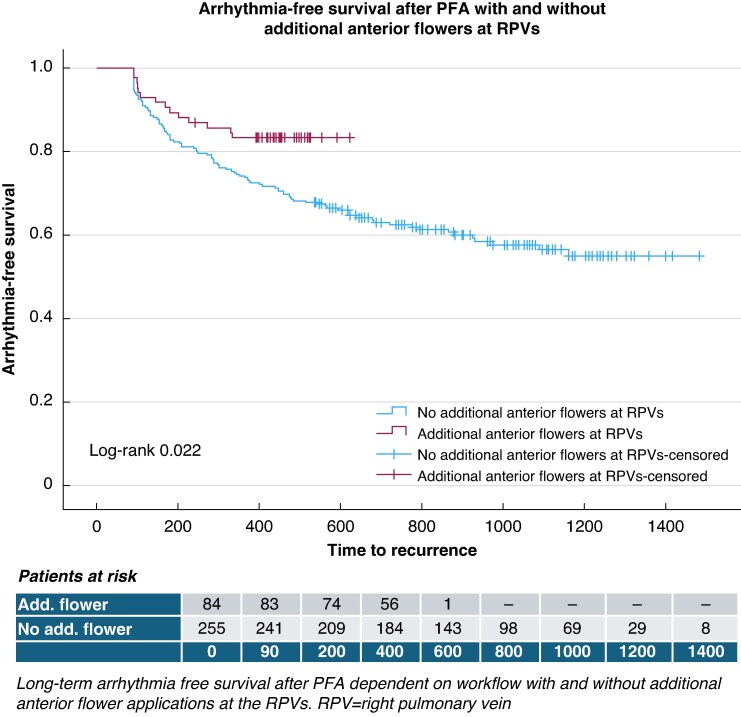
Success rate after first PVI in paroxysmal and persistent AF with and without additional anterior flower applications at the RPVs. PFA, pulsed field ablation; RPV, right pulmonary vein; PVI, pulmonary vein isolation; AF, atrial fibrillation.

During a median FUP period of 752 (391–1486) days, arrhythmia recurrence occurred in 63/214 PAF and 53/125 PersAF patients, corresponding to success rates of 71 and 58%, respectively, with a median time to recurrence of 218 (90–1161) days (*Figure 1A*, left). Recurrence occurred in 25% at 1 year, rising to 45% at 3 years of FUP. Analysis of multiple procedure outcome estimates improved long-term arrhythmia-free survival after repeat ablations, with an overall success rate of 86% (*Figure 1A*, right). Additional anterior flower applications at the right pulmonary veins (RPVs) during the first procedure led to an 83% arrhythmia-free survival during a FUP period up to 600 days (*Figure 2*).

### Predictors of arrhythmia recurrence full cohort

**Figure 3:**
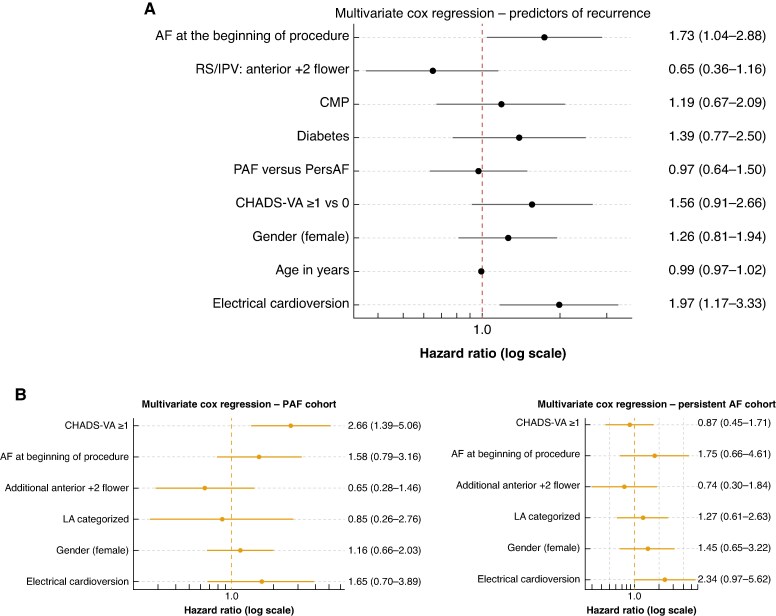
(*A*) Multivariate analysis of predictors for AF recurrence in the overall cohort. Variables that were statistically significant in the univariate analysis, as well as those considered clinically relevant, were included in a Cox regression model. (*B*) Multivariate Cox regression in patients with paroxysmal and in patients with persistent AF. A CHADS-VA score of 0 was significantly protective for maintaining sinus rhythm after AF ablation in the PAF-cohort. AF, atrial fibrillation; RS/IPV, right superior/inferior pulmonary vein; CMP, cardiomyopathy; PAF, paroxysmal atrial fibrillation; PersAF, persistent atrial fibrillation; LA, left atrium.

Multivariate analysis showed ECV at the end of the first procedure [HR 1.97 (95% CI 1.17–3.33), *P* = 0.01] and AF at the beginning of the procedure [HR 1.73 (95% CI 1.04–2.88); *P* = 0.03] being independently associated with a 2- and 1.7-times higher risk of arrhythmia recurrence. Additional anterior lesions at the RPVs, which seemed to be protective in the univariate analysis, showed only a protective trend [HR 0.65 (95% CI 0.36–1.16), *P* = 0.14] in the multivariate analysis (*Figure 3A*).

### Predictors of arrhythmia recurrence in paroxysmal and persistent AF

The multivariate Cox regression model of the PERS cohort did not show any statistically significant predictors associated with AF recurrence. CHADS-VA ≥ 1 was significantly associated with higher long-term AF recurrence in PAF [HR 2.66 (95% CI 1.39–5.06), *P* = 0.003], indicating a different predictive value across AF types. This result was confirmed in uni- and multivariate Cox models with interaction terms to assess whether the potential predictors for the recurrence of AF differed between PAF and PersAF [HR 0.34 (95% CI 0.16–0.75); *P* = 0.008]. Additional anterior flower applications showed a higher protective trend towards less recurrence in the PAF than the PERS cohort (*Table 3*; *Figure 3B*).

**Table 3:** Predictors of AF recurrence—patients with PAF vs. PersAF

	Paroxysmal atrial fibrillation (*n* = 214)			Persistent atrial fibrillation (*n* = 125)		
	Recurrence (*n* = 63)	No recurrence (*n* = 151)	*P*-value (univariate Cox regression)	Recurrence (*n* = 53)	No recurrence (*n* = 72)	*P*-value (univariate Cox regression)
**Gender**			***P*** **=** **0.044**			*P* = 0.376
Female	48%	33%		26%	17%	
Male	52%	67%		74%	83%	
**Age (year)**			*P* = 0.125			*P* = 0.922
Median (range)	63 (39–77)	62 (31–85)		62 (32–82)	63 (32–77)	
**LVEF**			*P* = 0.286			*P* = 0.834
≥50%	98%	94%		82%	86%	
49–41%	2%	3%		4%	4%	
40–35%	—	1%		10%	3%	
<35%	—	2%		4%	7%	
**LA diameter**				76%	84%	
Normal/mild/moderate severe/very severe dilation (≥50 mm)	94.7%	93%	*P* = 0.735			*P* = 0.328
	5.3%	7%		24%	16%	
**CHADS-VA**			***P*** **<** **0.001**			*P* = 0.931
Median (range)	2 (0–6)	1 (0–6)		2 (0–5)	2 (0–5)	
0	25%	55%		45%	46%	
≥1	75%	45%		55%	54%	
**BMI (kg/m^2^)**			*P* = 0.732			*P* = 0.556
Median (range)	27 (20–46)	26.5 (15–46)		28 (21–42)	28.5 (20–40)	
**Comorbidities**						
Hypertension	63.5%	54%	*P* = 0.264	58.5%	54%	*P* = 0.750
Diabetes	14%	5%	*P* = 0.014	11%	8%	*P* = 0.391
CAD	14%	17%	*P* = 0.837	17%	19%	*P* = 0.444
Hyperlipidaemia	27%	25%	*P* = 0.493	28%	31%	*P* = 0.771
Stroke	8%	7%	*P* = 0.625	7.5%	4%	*P* = 0.422
Cardiomyopathy	9.5%	6%	*P* = 0.252	23%	15%	*P* = 0.158
**Anatomical variant**			*P* = 0.634			*P* = 0.583
Normal anatomy	78%	74%		77%	74%	
Anatomical variant	22%	26%		23%	26%	
**NT-proBNP (pg/mL)**			*P* = 0.591	544 (21–2258)	294 (13–4696)	*P* = 0.583
Median (range)	192.5 (39–2165)	129.5 (6–2413)				
**Creatinine (mg/dL)**			*P* = 0.624			*P* = 0.243
Median (range)	0.96 (0.54–1.69)	0.98 (0.62–1.88)		1.11 (0.77–1,.84)	1.05 (0.7–1.36)	
**First pass isolation**	89%	83%	*P* = 0.372	79%	74%	*P* = 0.872
**Additional anterior flowers (%)**	13%	30%	***P*** **=** **0.081**	11%	35%	*P* = 0.166
**Catheter size**			*P* = 0.973			*P* = 0.650
31 mm	98%	98.5%		92%	92%	
35 mm	2%	1.5%		8%	8%	
**Procedure duration**			*P* = 0.512			*P* = 0.697
Median (range)	56 (27–128)	52 (24–167)		60.5 (43–134)	55 (31–175)	
**X-ray dose**	10.4 (0.18–54.6)	11.4 (0.81–110)	*P* = 0.979	15 (3.3–60.8)	15 (2.6–101)	*P* = 0.581
Median (range)						
**Heart rhythm at the beginning of the proc.**			***P*** **=** **0.009**			***P*** **<** **0.001**
SR or pacing	71%	87%		23.5%	59%	
AF	29%	13%		76.5%	41%	
**ECV at the end**	14.5%	7%	***P*** **=** **0.042**	63.5%	29%	***P*** **<** **0.001**
Yes						

LVEF, left ventricular ejection fraction; LA diameter, left atrial diameter (measured in PLAX view); BMI, body mass index; AF, atrial fibrillation; PAF, paroxysmal AF; PersAF, persistent AF; CAD, coronary artery disease; SR, sinus rhythm; ECV, electrical cardioversion; — none.

Values in brackets show range (minimum–maximum).

## Reconnections of PVs and additional ablation strategies during redo procedures

**Figure 4:**
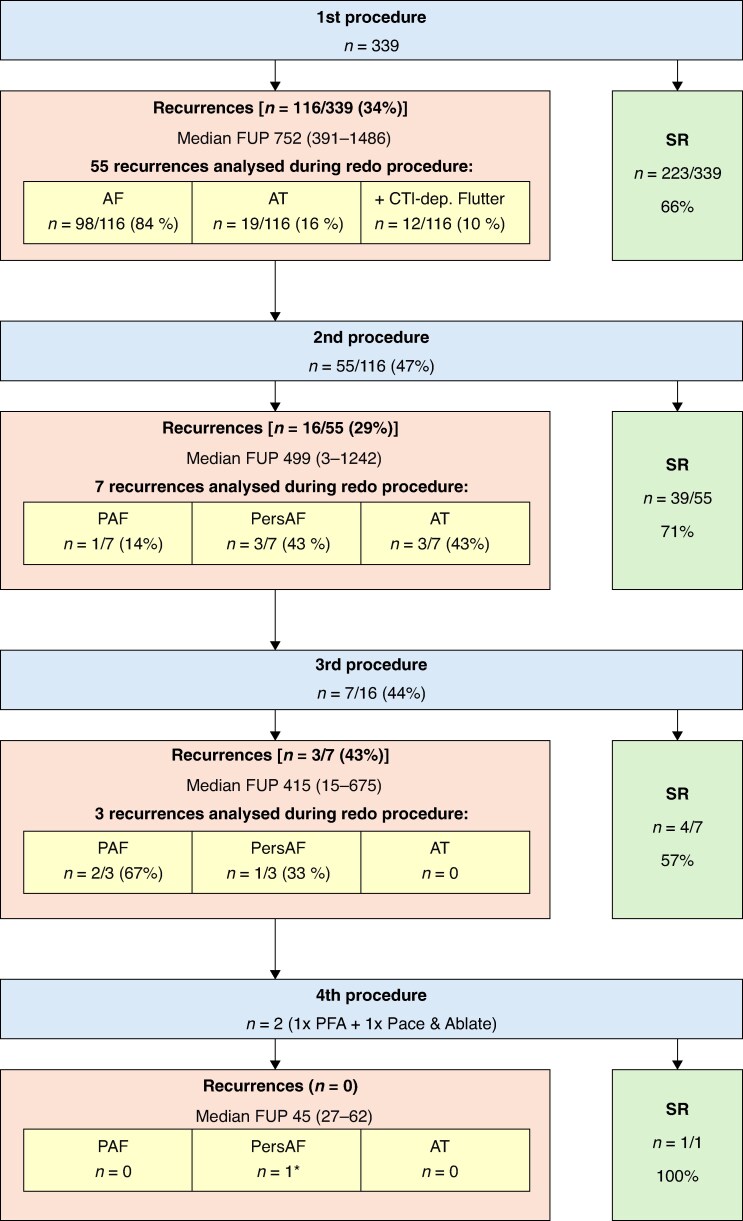
Incidence and types of recurrences after repeat ablation. Median FUP—values in brackets show range (minimum–maximum). FUP, follow-up; AF, atrial fibrillation; AT, atrial tachycardia; LMRAT, left macroreentrant atrial tachycardia; FAT, focal atrial tachycardia; CTI, cavotricuspid isthmus; SR, sinus rhythm; PAF, paroxysmal atrial fibrillation; PersAF, persistent atrial fibrillation; PFA, pulsed field ablation. *Patient after pace and ablate strategy.

**Figure 5:**
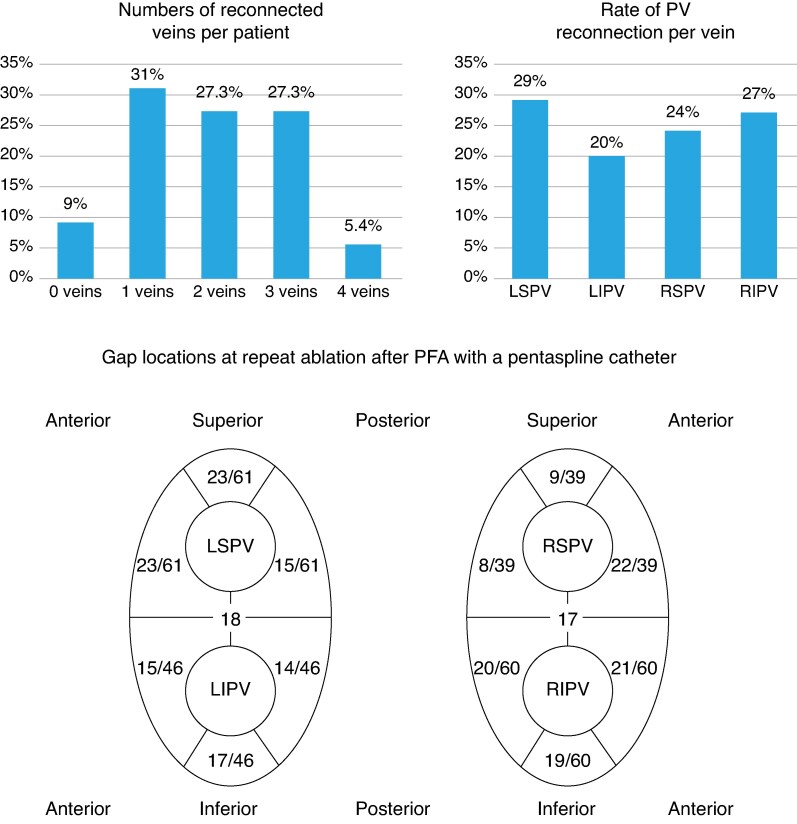
Total numbers of reconnected veins on a per-patient basis. In most cases one vein showed gaps (31%), and two or three veins were reconnected in 27.3%. In only 9% no gaps could be detected at the redo procedure, and in 5.4% of cases all four veins were reconnected. Rate of pulmonary vein reconnections per vein: the highest incidence for reconnections could be shown for the LSPV (29%), directly followed by the right pulmonary veins (RIPV 27%, RSPV 24%). The lowest rate of gaps was seen at the LIPV (20%). In total, 47% of veins showed gaps at the redo procedure. The LSPVs showed 61 gaps, whereas most of them were located at the anterior-superior aspect. The LIPVs showed 46 gaps, most of them inferiorly. All RSPVs showed 39 gaps, the great majority of them at the anterior aspect, which was also the case at the RIPV with 60 gaps in total and most of them at the anterior aspect. PV, pulmonary vein; LSPV, left superior pulmonary vein; LIPV, left inferior pulmonary vein; RSPV, right superior pulmonary vein; RIPV, right inferior pulmonary vein.

**Figure 6:**
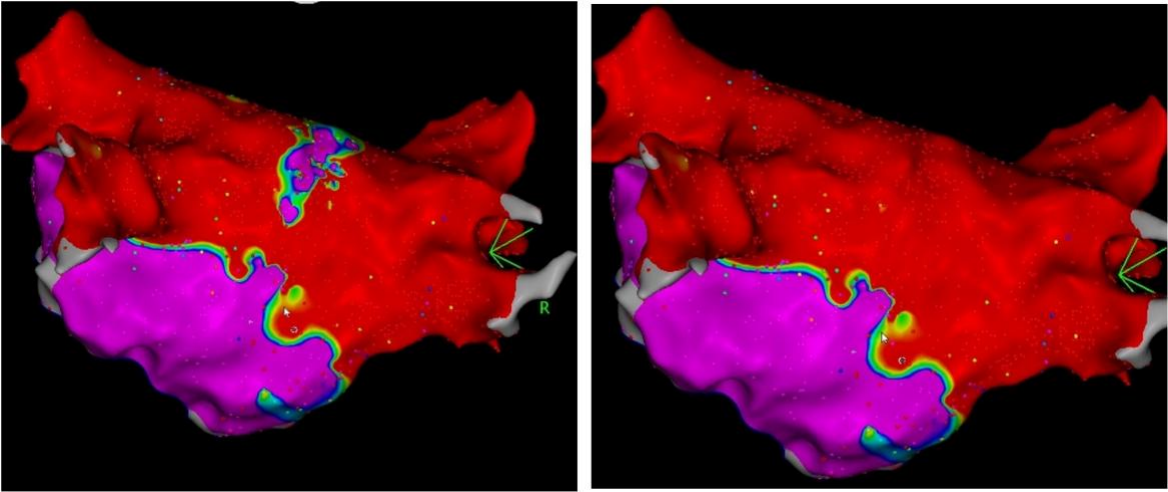
Posterior wall isolation due to a narrow remaining corridor after PFA.

In 116 patients with arrhythmia recurrences, 84% of patients suffered from AF, while only 16% showed AT. *Figure 4* shows the incidence and type of recurrences after redo procedures. During the redo procedure, 104 out of 221 veins were reconnected (47%). On a per-patient level, 0–4 veins were reconnected as shown in *Figure 5*. All RMPVs were still isolated. Using a mixed-effects logistic regression model, restricted to the four standard PVs, no significant differences in reconnection rates across all PVs could be found. Right superior pulmonary vein (RSPV), left superior pulmonary vein (LSPV), and right inferior pulmonary vein (RIPV) were reconnected most often at the anterior aspect (88%, *n* = 22/25/77%, *n* = 23/30/75%, *n* = 21/28). However, the LSPV showed the same number of gaps at the superior aspect (77%, *n* = 23/30). Upon reconnections of the left inferior pulmonary vein (LIPV), most gaps were observed inferiorly (81%, *n* = 17/21). In dichotomized McNemar analysis, anterior segments showed significantly more gaps than posterior segments (*P* = 0.049). All reconnected veins were successfully reablated during the redo procedure. Additional ablation strategies included CTI (1st/2nd procedure: 5.6%/24%), PWI (2%/15%), MI line (2%/15%), and roof line (0%/7%). In five of eight cases (63%), we performed PWI due to an incomplete unintentionally isolated posterior wall during the index procedure (*Figure 6*). Of these five patients, only one showed roof-dependent LMRAT.

### Third procedure

Sixteen patients experienced another arrhythmia recurrence, of whom seven underwent re-redo procedures. We performed four cases with RF, two with Farapulse® and one with Affera®. In four cases, none of the PVs were reconnected (57%). PWI was performed in four patients (one due to an only very thin remaining isthmus), MI line in two patients (both reconnected), and reconnected roof line in another patient as the ablation strategy. In two cases we had to isolate the CTI (one reconnected). Further information relating to the 3rd and 4th procedures is shown in the supplements.

## Discussion

To our knowledge this is the first study providing long-term FUP to a maximum of more than 3 years after PFA with a pentaspline catheter and analysing multiple procedure outcomes. Key findings are low AT recurrence rates after the first procedure, high PV reconnection rates in redo patients, and additional anterior lesions at the RPVs showing a trend towards protection against recurrences. Most importantly, 71% of patients with PAF remained in SR for up to 3 years after the initial ablation. Furthermore, an increase in success rate was documented after a multiple procedure approach to 86%, irrespective of whether PAF or PersAF.

## Predictors of arrhythmia recurrence

Approximately 20–50% of patients experience recurrence at 5 years.^12^ Not surprisingly, presentation in AF at the beginning and the need for ECV at the end of the procedure were related to recurrence. Severely enlarged LA size, a known predictor for AF recurrence,^13,14^ was not associated with recurrence in our cohort. This may be limited by the fact that 43% had normal LA size, while only 10 and 2% showed severely or very severely dilated atria, respectively. This can be well explained by the local SOP, restricting AF ablation to those with a realistic success rate, offering PVI in severely dilated LA only in the setting of advanced heart failure. We found that CHADS-VA ≥ 1 was significantly associated with higher long-term AF recurrence in PAF rather than PersAF. This finding probably highlights that recurrence mechanisms in PAF are more strongly influenced by clinical risk profile, whereas in PersAF the advanced atrial substrate dominates recurrence risk.

Another open question is whether early recurrence (ER) during the BP of 90 days predicts late recurrence (LR). We didn’t screen for ER, as the first Holter ECG was performed only after 3 months. Nevertheless, in 31% of patients with LR, ER was also documented. Several mechanisms can be recognized for ER after thermal ablation, such as acute inflammatory changes, ischaemia, myocardial necrosis, and oxidative stress.^2^ Given that these mechanisms are not present in patients after PFA,^15^ the optimal duration of the BP and implication of ER should be further assessed, as others also suggest.^16^

Our findings on short-term success rates align with recent pooled real-world analyses^17,18^ and other redo data.^19–23^ We could show that even with a median FUP of 752 days, single-procedure success reached 71% in PAF and 59% in PersAF patients. Hence, outcomes beyond 12 months also seem comparable to thermal methods. Notably, in a single-centre analysis of 400 patients after high-power short-duration (HPSD) ablation, only 3% suffered from AF recurrence, and 11 out of 12 patients had chronically isolated PVs at redo procedures.^24^ Kassa *et al*.^25^ found that patients after HPSD show complete PVI in redo procedures significantly more often than those after low-power low-duration or cryoballoon ablation. Comparison of reconnection patterns in patients after PFA or HPSD ablation could be of interest.

### PV reconnections

#### Acute PV reconnections at index procedure

The optimal timing for assessment of acute PV reconnections remains to be determined, as due to PV stunning, it is not clear whether assessment during the procedure is useful at all. Mills *et al*.^26^ recommend the adoption of a standardized ‘waiting period’ before assessing complete PV isolation, without specification of an exact time frame. Most studies implemented a 20 min waiting period, reporting acute reconnection rates of 4%,^3^ 0%,^27^ and 1.4%,^22^ among others. Persistent signals likely indicate incomplete isolation, justifying additional lesions, with remapping potentially providing valuable supplementary information. However, Kueffer *et al*.^28^ demonstrated the reliability of the PFA catheter in assessing PVI in the absence of a defined waiting period. Reliability can even be enhanced when adapting the pacing output threshold to avoid high-output pace capture, a consequence of the catheter’s design. Our findings suggest that the application of additional pulses, due to missing first pass isolation (FPI), does not influence long-term recurrence. However, we could show that acute PV reconnection is present the most often at the LSPV, which aligns with findings from other authors.^26,29^

#### PV reconnections at redo procedures

In our cohort we found reconnections at 47% of all veins. Only 9% of patients (5/55) showed no reconnected PV. Others have observed a significantly higher percentage of durably isolated PVs.^10,19,20,22,30,31^ This could be due to the fact that we did not only count missing exit block but also gaps according to high-density bipolar voltage mapping to be a clinically relevant PV reconnection. It could also be explained by the relatively long time period to the redo procedure [9 (6–19) months] compared to others (6.1 ± 4 months)^24^/[7 (5–10) months].^20^ Among all the redo papers published to date, the case where all veins showed reconnections was very rare (0–7.6%).^5,10,19,20,32^

In our cohort, LSPV (29%) was the most often acutely and chronically reconnected, consistent with previous findings,^23,26,29,30^ emphasizing the need for us to pay more attention to fully isolating this vein on the first attempt. Other authors described RIPV^10,19,31–33^ and RSPV to be the most commonly reconnected,^22^ resembling our cohort, where RIPV (27%) and RSPV (24%) were reconnected the second most often. We assume that RIPV is commonly reconnected because of difficult anatomical accessibility.^34^

#### Frequent sites of reconnections and ideas for improvement

It is of paramount importance to identify solutions that can prevent PV reconnection and improve our PFA results. PFA with a pentaspline catheter is fast and safe, yet optimal efficacy requires precise technique, tailored lesion delivery, and further insights into lesion durability and biophysics.

Anterior PV reconnections predominated, except in the LIPV, where gaps were mainly inferior. However, frequent gaps at the anterior aspect were described by many authors.^19,20,33^ In 2023, Ruwald *et al*.^32^ published their findings on anterior gaps at the right PVs, leading to the adoption of additional anterior applications to the RPVs, which were performed in 25% of patients in this cohort and showed a protective effect for arrhythmia recurrence. We suggest that this specific predictor should be investigated again in a cohort with a higher number of patients with uniform FUP durations. Schaack *et al*.^33^ demonstrated that additional olive-shaped applications to each vein reduced PV reconnections, but did not improve arrhythmia-free survival in a propensity score-matched analysis. Evidence remains insufficient to recommend routine additional distal ostial or anterior RPV lesions. Nowadays many EP laboratories have developed their own PFA workflow; a global protocol is still missing. Of course, all these approaches need to be further evaluated.

Besides repetition dependence, tissue contact appears to be one of the most important factors to create durable PF lesions and to reduce lesion retraction probability, as shown by various authors.^35–37^ Currently available integrated mapping systems do not allow for precise anatomical mapping. The total procedure and left atrial dwell time are significantly longer, but 3D-EAM appears to reduce fluoroscopy time^38^ and improve contact, pending confirmation in randomized trials. Without preprocedural imaging, intraprocedural mapping, or intracardiac echocardiography (ICE), veins may be missed. Additionally, the use of ICE allows for guiding to precise positioning of the catheter at the PV ostia, enhancing contact.^39^ 3D-EAM can help avoid ablation of wide areas, especially in small atria, often leading to thin remaining corridors at the posterior wall, as we and others demonstrated.^20,23,29,32^ This should be avoided, even if some conclude that incomplete PWI does not lead to dysrhythmia recurrence in most cases.^40^

## Limitations

The results of this study are derived from a single centre and are observational, with data being analysed retrospectively, therefore non-randomized. The multiple procedure analysis is subject to survivor bias, as only patients with recurrence and sufficient FUP to undergo repeat ablation were included, with FUP reset after the final ablation.

## Conclusion

In patients treated with PFA for paroxysmal or persistent AF, PV reconnections were frequent in patients presenting for repeat ablations. In a FUP of around 2 years, multiple procedure approach resulted in arrhythmia-free survival in 86% of patients. Preselection of patients for first-do PVI is crucial to ensure low recurrence rates and to select the right technology and workflow for each individual patient.

## Supplementary Material

euag057_Supplementary_Data

## Data Availability

The data underlying this article are available within the article and its supplementary material. Additional details may be available from the corresponding author on request.
