# Supplementary figures and images for: Incidence and predictors of AF recurrence during long-term follow-up of patients after PF ablation for atrial fibrillation
Source: Europace. 2026 Mar 23;28(4):euag057. doi: 10.1093/europace/euag057 (PMC13122355; doi:10.1093/europace/euag057)

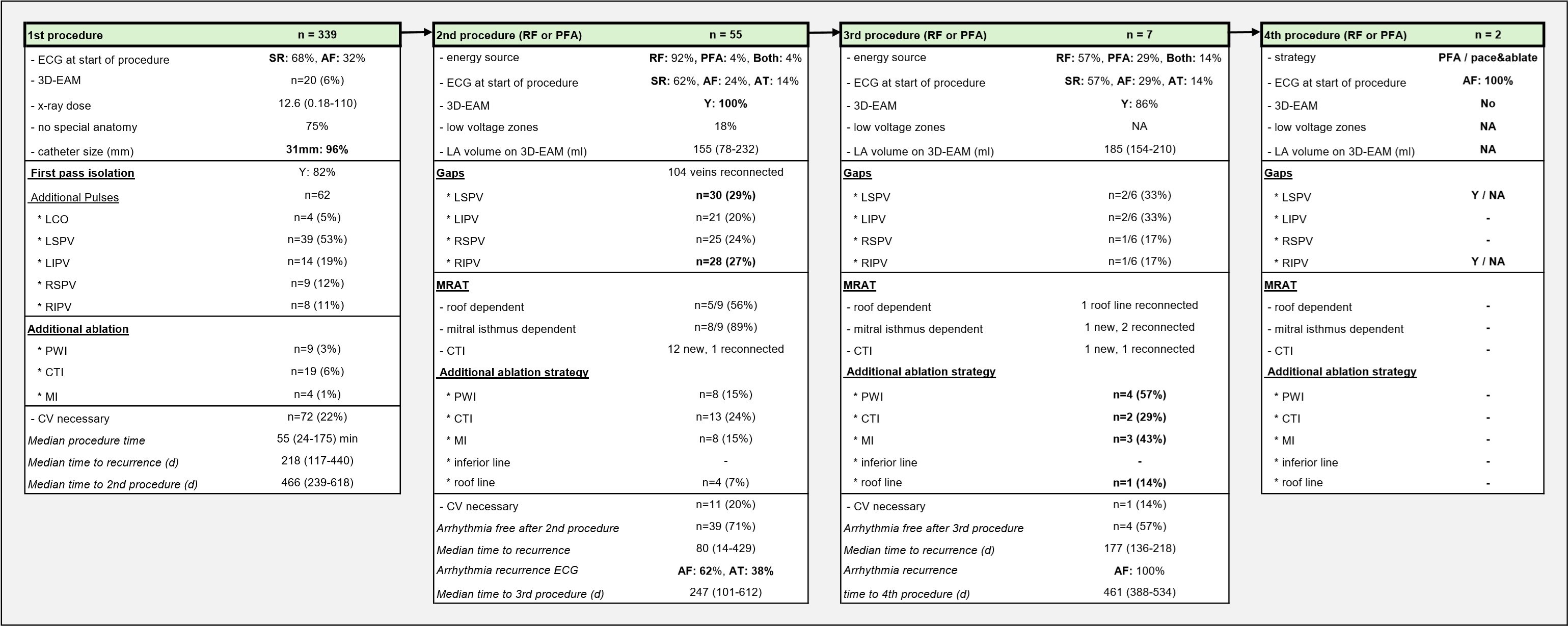

Supplement: euag057_Supplementary_Data [file euag057_supplementary_data.jpeg]
